# Prospective ultrasonographic phenotyping and infectious polymerase chain reaction screening of canine hepatobiliary disease in southern Vietnam

**DOI:** 10.14202/vetworld.2026.2479-2495

**Published:** 2026-06-20

**Authors:** Tran Thi Thao, Nguyen Tran Phuoc Chien, Tran Ngoc Bich

**Affiliations:** Faculty of Veterinary Medicine, College of Agriculture, Can Tho University, 3/2 Street, Ninh Kieu Ward, Can Tho City 94000, Vietnam

**Keywords:** canine adenovirus type 1, cirrhosis, hepatic lipidosis, hepatobiliary disease, hepatitis, polymerase chain reaction, ultrasonography, vector-borne pathogens

## Abstract

**Background and Aim:**

Canine hepatobiliary disorders are frequently encountered in small animal practice; however, prospective clinic-based studies integrating ultrasonographic phenotyping with infectious molecular screening remain limited in tropical Southeast Asia. This study aimed to determine the frequency of ultrasonographically detected hepatobiliary abnormalities, identify associated host factors, characterize ultrasonographic phenotypes, and assess infectious etiologies in dogs with suspected hepatobiliary disease in southern Vietnam.

**Materials and Methods:**

A prospective observational study was conducted at the Veterinary Teaching Clinic, Can Tho University, Vietnam, between January and November 2025. Among 1,278 canine presentations, 111 dogs meeting predefined clinical and clinicopathologic criteria underwent abdominal ultrasonography, and 63 dogs with confirmed hepatobiliary abnormalities were enrolled. Hematologic and serum biochemical analyses were performed in all enrolled dogs. Cases classified with a hepatitis ultrasonographic phenotype underwent conventional polymerase chain reaction (PCR) screening of ethylenediaminetetraacetic acid (EDTA)- anticoagulated blood samples targeting vector-borne pathogens, *Leptospira* spp., and canine adenovirus type 1. Ultrasonographic phenotypes were categorized as hepatitis, hepatic lipidosis, or cirrhosis. Univariable logistic regression was used to evaluate host-associated risk factors, and hierarchical clustering analysis was applied to assess co-occurring clinical and imaging abnormalities.

**Results:**

Hepatobiliary abnormalities were identified in 63/1,278 dogs (4.93%) and in 63/111 clinically suspected dogs (56.76%). Ultrasonographic positivity was significantly associated with exotic breed (odds ratio [OR] = 2.29), age >8 years (OR = 13.93), obesity (OR = 4.78), and underweight body condition (OR = 3.38). Hepatitis was the predominant ultrasonographic phenotype (57.14%), followed by hepatic lipidosis (30.16%) and cirrhosis (12.70%). Dogs with hepatitis commonly exhibited anorexia, vomiting, diffuse hepatic hypoechogenicity, hepatomegaly, and gallbladder abnormalities. PCR screening detected infectious agents in 55.56% of hepatitis cases, including vector-borne pathogens (41.67%), *Leptospira* spp. (8.33%), and canine adenovirus type 1 (5.56%). Dogs with hepatic lipidosis showed diffuse hepatic hyperechogenicity and hepatomegaly, whereas cirrhosis cases demonstrated heterogeneous hepatic echotexture, ascites, and vascular abnormalities consistent with portal hypertension.

**Conclusion:**

Ultrasonography effectively identified distinct hepatobiliary imaging phenotypes in dogs and provided a practical diagnostic framework for referral-based clinical settings in resource-limited regions. Integration of ultrasonography with clinicopathologic evaluation and targeted PCR screening enhanced preliminary etiologic assessment, particularly in dogs with inflammatory hepatopathies. Older age and abnormal body condition were important host-associated factors linked with ultrasonographic positivity. Although imaging-based classification may assist clinical triage, histopathological confirmation remains necessary for definitive diagnosis.

## INTRODUCTION

Canine hepatobiliary disorders are common reasons for diagnostic evaluation in small animal practice and encompass a broad spectrum of inflammatory, metabolic, infectious, and degenerative conditions that frequently present with overlapping clinical signs and clinicopathologic abnormalities. Dogs affected by hepatobiliary disease commonly exhibit nonspecific clinical manifestations, including vomiting, anorexia, lethargy, and cranial abdominal discomfort, accompanied by variable alterations in hepatocellular and cholestatic biomarkers [1, 2]. Clinic-based studies from different regions have described the distribution of hepatic disorders and their associated clinicopathologic patterns; for example, investigations in the United Kingdom reported reactive hepatopathy and chronic hepatitis among the most frequently diagnosed conditions [1]. Despite these advances, accurate clinical classification remains challenging due to the heterogeneity of hepatobiliary disease and the limited specificity of noninvasive diagnostic tools.

Abdominal ultrasonography is widely used as a first-line imaging modality for evaluating hepatobiliary abnormalities in dogs because it provides a rapid, noninvasive assessment of the hepatic parenchyma, biliary structures, vascular architecture, and associated abdominal changes. Ultrasonography can identify characteristic imaging patterns suggestive of inflammatory, metabolic, or chronic degenerative liver disorders and may facilitate early clinical decision-making in referral and general practice settings. However, substantial overlap exists among ultrasonographic findings associated with different hepatopathies, and interpretation often requires integration with clinicopathologic and etiologic investigations. Consequently, the diagnostic value of ultrasonography may be enhanced when combined with targeted molecular testing and comprehensive clinical assessment.

In Vietnam and other tropical Southeast Asian regions, important knowledge gaps persist regarding the clinic-based epidemiology and phenotypic characterization of canine hepatobiliary disease. Available studies are largely retrospective or descriptive and primarily focus on clinical or biochemical findings without integrating standardized ultrasonographic phenotyping or molecular diagnostic approaches [3–5]. Furthermore, infectious hepatitides and vector-borne diseases are highly prevalent in tropical environments and may produce hepatobiliary injury patterns that overlap with primary liver disorders, thereby complicating clinical interpretation in the absence of etiologic testing. In this context, the selection of polymerase chain reaction (PCR) targets in the present study was guided by regional epidemiological relevance and clinical applicability. Vector-borne pathogens are commonly encountered in Vietnam and are frequently associated with systemic infections involving hepatic dysfunction. *Leptospira* spp. represent a major cause of acute hepatocellular injury and zoonotic disease in Southeast Asia, often presenting with overlapping hepatic and renal manifestations. Canine adenovirus type 1 (CAdV-1), although less frequently reported in routine clinical settings, remains a recognized etiologic agent of infectious canine hepatitis. These targets were therefore selected to represent clinically relevant infectious differentials that can be assessed using noninvasive, blood-based PCR in a referral clinical setting, where invasive diagnostic procedures such as liver biopsy are not routinely performed. This targeted approach provides a pragmatic balance between diagnostic yield and feasibility in resource-limited veterinary practice.

Despite increasing recognition of canine hepatobiliary disorders in companion animal medicine, substantial limitations remain in the current understanding of their clinic-based occurrence, imaging phenotypes, and infectious associations in tropical Southeast Asian regions. Most available studies from Vietnam have relied on retrospective case reviews or descriptive clinical observations, with limited incorporation of standardized ultrasonographic classification systems and molecular diagnostic techniques [3–5]. Consequently, there is insufficient prospective evidence describing how ultrasonographic abnormalities correlate with clinical manifestations, clinicopathologic alterations, and infectious etiologies in dogs with suspected hepatobiliary disease. In addition, the contribution of infectious agents, particularly vector-borne pathogens, *Leptospira* spp., and CAdV-1, to hepatobiliary abnormalities in referral hospital populations remains poorly characterized in Vietnam. The absence of integrated diagnostic frameworks that combine ultrasonography, clinicopathologic evaluation, and PCR-based etiologic screening has limited the development of practical, evidence-based diagnostic approaches in resource-constrained veterinary settings.

Another important gap involves the lack of phenotype-driven classification models applicable to routine clinical practice in regions where advanced diagnostics such as histopathology or immunohistochemistry are not readily accessible. Although ultrasonography is frequently used in small animal medicine, standardized characterization of ultrasonographic phenotypes associated with hepatitis, hepatic lipidosis, and cirrhosis has not been comprehensively evaluated in prospective canine populations in southern Vietnam. Furthermore, limited data are available regarding host-associated factors that may influence ultrasonographic positivity in clinically suspected cases. Addressing these gaps is essential to improve early recognition, clinical triage, and preliminary etiologic assessment of canine hepatobiliary disease in tropical veterinary practice.

This study aimed to estimate the clinic-based frequency of ultrasonographically detected hepatobiliary abnormalities in dogs presented to a tertiary Veterinary Teaching Clinic in southern Vietnam and to characterize the associated ultrasonographic phenotypes. The study further aimed to evaluate host-associated risk factors linked with ultrasonographic positivity among clinically suspected dogs and to investigate selected infectious etiologies using blood-based PCR in dogs exhibiting a hepatitis ultrasonographic phenotype.

In addition, this study sought to develop a clinically applicable, phenotype-driven diagnostic framework integrating ultrasonography, clinicopathologic evaluation, and targeted PCR screening for use in resource-limited veterinary settings. By prospectively combining imaging findings with molecular etiologic assessment, this study aimed to provide contemporary regional data to improve the preliminary classification and clinical management of canine hepatobiliary disease in tropical Southeast Asia.

## MATERIALS AND METHODS

### Ethical approval

Ethical approval for this study was granted by the Animal Ethics Committee of Can Tho University, Vietnam (CTU-AEC26001). The study involved client-owned dogs presented to the Veterinary Teaching Clinic, Can Tho University, Vietnam, for routine clinical evaluation and diagnostic investigation. No experimental infection, unnecessary invasive intervention, or non-therapeutic procedure was performed during the study period. All diagnostic procedures, including abdominal ultrasonography, blood collection, hematologic analysis, serum biochemical testing, and PCR-based screening, were conducted in accordance with standard veterinary clinical practice guidelines to minimize stress and discomfort in the animals.

Written informed consent was obtained from all dog owners before enrollment. Owners were informed about the objectives of the study, sample collection procedures, the use of anonymized clinical data, and the publication of ultrasonographic images for scientific purposes. Participation was voluntary, and all collected data were handled confidentially in accordance with institutional ethical standards.

### Study period and location

This prospective, hospital-based observational study was conducted between January and November 2025 at the Veterinary Teaching Clinic, College of Agriculture, Can Tho University, Vietnam. The clinic functions as a tertiary referral center receiving both first-opinion and referred canine cases from urban and peri-urban areas within the Mekong Delta region.

### Study design and animal selection

All client-owned dogs presented to the Veterinary Teaching Clinic during the study period (n = 1,278 presentations) were screened for eligibility. Dogs were classified as having suspected hepatobiliary disease if they fulfilled at least one clinical criterion, including vomiting, diarrhea, anorexia, lethargy, icterus, cranial abdominal pain, abdominal distension/ascites, or suspected hepatomegaly, together with at least one clinicopathologic abnormality, including increased alanine aminotransferase (ALT), aspartate aminotransferase (AST), alkaline phosphatase (ALP), hyperbilirubinemia, or hypoalbuminemia. Neonatal puppies aged <2 months and dogs lacking essential clinical, ultrasonographic, or laboratory data were excluded from the study.

Sample size was determined based on all eligible cases presented during the study period, and no *a priori* power calculation was performed because of the exploratory and clinic-based nature of the investigation. This study design provided a pragmatic framework for imaging-based phenotypic classification in settings where histopathology is infrequently available and where accessible imaging and molecular diagnostic tools are more clinically feasible.

### Abdominal ultrasonography and image interpretation

Abdominal ultrasonography was performed using an ultrasound system (Mindray DP10VET, Mindray Bio-Medical Electronics Co., Ltd., Shenzhen, China) by a single clinician with >5 years of experience in small animal diagnostic imaging to minimize interobserver variability. Dogs were fasted for at least 8 h before examination whenever clinically feasible.

A microconvex probe (5–10 MHz) was used with standardized gain and depth settings. Dogs were examined in dorsal and lateral recumbency. The liver was evaluated for size, echogenicity, echotexture, hepatic margins, and vascular structures. Heterogeneous echotexture was defined as nonuniform parenchymal echogenicity, and blunt margins were defined as rounded hepatic edges. Hepatic hypoechogenicity/hyperechogenicity was assessed relative to the renal cortex.

Gallbladder wall thickening was defined as >2.0 mm, and gallbladder volume (VGB) was estimated using the ellipsoid formula:


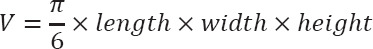


The calculated value was normalized to body weight (BW; mL/kg) [6, 7]. Ultrasonographic findings were recorded as binary variables (present/absent) for phenotyping analyses (Table 1) [6, 7].

**Table 1:** Ultrasonographic parameters used for canine liver assessment [6, 7].

Parameter	Physiological feature	Normal value	Abnormal changes and diagnostic significance
L–K ratio (liver-to-kidney echogenicity ratio)	Comparison of hepatic echogenicity with the renal cortex	Liver is equal to or slightly more echogenic than the renal cortex	Decreased hepatic echogenicity suggests hepatitis; increased echogenicity is consistent with hepatic lipidosis and hepatic fibrosis/cirrhosis
Hepatic parenchymal echogenicity	Parenchymal homogeneity and echogenicity intensity	Homogeneous, medium echogenicity	Diffuse hypoechogenicity indicates inflammation or congestion; diffuse hyperechogenicity suggests fatty change and fibrosis; heterogeneous echotexture may indicate neoplasia, cysts, or abscesses
Gallbladder volume (V_GB_)	Volume of bile contained in the gallbladder at a given time point	< 1 mL/kg	Markedly increased volume may be associated with biliary obstruction or chronic hepatobiliary disease
Gallbladder wall thickness	Thickness of the gallbladder wall at the thinnest point	≤ 1.5–2.0 mm	Wall thickness > 2 mm suggests cholecystitis; increased wall echogenicity may indicate fibrosis; a thickened wall with abundant sludge is consistent with cholestasis secondary to cholelithiasis (cholecystitis)
Portal vein diameter (dPV)	Measured at the hepatic hilus	3.3–10.5 mm	< 3.3 mm may reflect decreased hepatic perfusion, prolonged fasting, hepatic hypoplasia, or hypotension; > 10.5 mm suggests portal hypertension
Ascites (peritoneal fluid)	Anechoic fluid around the liver/gallbladder	Absent	Anechoic transudate indicates non-inflammatory effusion; finely echogenic exudate indicates inflammatory effusion. May result from portal hypertension, peritonitis, or hepatic failure

L = liver, K = kidney, PV = portal vein, V_GB_ = gallbladder volume.

### Ultrasonographic case definitions

Dogs with ultrasonographic evidence of liver disease were classified into three imaging-based phenotypes (imaging-supported rather than biopsy-confirmed etiologic diagnoses). The hepatitis phenotype was characterized by diffuse parenchymal changes dominated by hypoechogenicity and heterogeneous echotexture, frequently accompanied by hepatomegaly and blunt hepatic margins, with or without hepatic venous dilation; concurrent gallbladder abnormalities were also possible [6, 7]. The hepatic lipidosis phenotype was defined by diffuse hepatic echogenicity, increased liver-to-renal cortical echogenicity, and reduced conspicuity of intrahepatic and portal vasculature, often accompanied by hepatomegaly. Severity grading (grades 1–3) was performed according to published ultrasonographic criteria [7, 8].

The cirrhosis phenotype was characterized by diffuse, heterogeneous echotexture, an atrophic liver, and irregular hepatic margins, frequently associated with peritoneal effusion and hepatic venous dilation [6, 7, 9].

These imaging-based classifications were interpreted as clinical phenotypes rather than definitive diagnoses, as histopathological confirmation was not obtained. Liver biopsy was not conducted because of clinical constraints, including cost, owner consent, and the invasive nature of the procedure in a routine clinical setting. Table 2 summarizes the ultrasonographic phenotypes identified in this study [7–9].

**Table 2:** Ultrasonographic phenotypic classification of hepatobiliary disorders in dogs.

Disorder	Ultrasonographic features	Pathophysiologic mechanism
Hepatitis	Mild hepatomegaly; decreased echogenicity in acute hepatitis and increased echogenicity in chronic hepatitis; reduced parenchymal uniformity. Vascular margins are more conspicuous in acute hepatitis but become less conspicuous in chronic hepatitis. Mild ascites may be present [7].	Acute inflammation causes congestion and inflammatory cell infiltration, increasing tissue water content, which results in hypoechogenicity.
Hepatic lipidosis	Grade 1: mild diffuse increased hepatic echogenicity; diaphragm and portal vein walls are easily visualized [8].	Lipid accumulation within hepatocytes increases ultrasound backscatter, leading to hyperechogenicity.
		Grade 2: moderate diffuse increased echogenicity with reduced definition of portal vein walls and the diaphragm [8].
		Grade 3: marked diffuse increased echogenicity with poor or absent visualization of portal vein walls, diaphragm, and the far field/posterior aspect of the right hepatic lobe [8].
Cirrhosis	Small liver; irregular margins; markedly increased echogenicity; heterogeneous architecture. Portal vein dilation; ascites may be present [9].	Fibrous tissue deposition causes hepatic contraction and increased echogenicity, and contributes to portal hypertension.

### Laboratory testing

Hematologic analysis was performed using an automated hematology analyzer (Dymind DF50, Dymind Biotechnology Co., Ltd., Shenzhen, China), and serum biochemical analysis was conducted using a biochemical analyzer (MNCHIP Pointcare M4, MNCHIP Technologies Co., Ltd., Tianjin, China) according to manufacturer-validated protocols for canine samples. Reference intervals were based on manufacturer guidelines and previously published canine reference values [10].

Blood samples were processed within 2 h after collection to maintain analytical stability. Internal quality-control procedures were routinely performed in accordance with standard laboratory protocols.

### PCR etiologic screening

For dogs exhibiting a hepatitis ultrasonographic phenotype, ethylenediaminetetraacetic acid-anticoagulated blood samples were subjected to conventional PCR targeting selected infectious agents, including vector-borne pathogens (*Ehrlichia canis*, *Anaplasma platys*, *Babesia* spp., and *Hepatozoon canis*), *Leptospira* spp., and CAdV-1 (Table 3) [11–16].

**Table 3:** Polymerase chain reaction primers used for the detection of selected canine infectious pathogens.

Target organism	Target gene	Primer sequence (5′–3′)	Amplicon size (bp)	Reference
*Ehrlichia canis*	*16S rRNA*	GAACGAACGCTGGCGGCAAGCC	478	[13]
		CGTATTACCGCGGCTGCTGGC		
		TATAGGTACCGTCATTATCTTCCCTAT	389	
		CAATTATTTATAGCCTCTGGCTATAGGAA		
*Anaplasma platys*	*16S rRNA*	GAACGAACGCTGGCGGCAAGCC	478	[15]
		CGTATTACCGCGGCTGCTGGC		
		TTTGTCGTAGCTTGCTATG	402	
		GAGTTTGCCGGGACTTCTTCT		
*Babesia* spp.	*18S rRNA*	GTTTCTGMCCCATCAGCTTGAC	420–440	[14]
		CAAGACAAAAGTCTGCTTGAAAC		
*Hepatozoon canis*	*18S rRNA*	ATACATGAGCAAAATCTCAAC	625	[16]
		CTTATTATTCCATGCTGCAG		
*Leptospira* spp.	*16S rRNA*	GCGCGTCTTAAACATGC AAG	307	[11]
		CTTAACTGCTGCCTCCCG		
CAdV-1	*L*	CGCGCTGAACATTACTACCTTGTC	508	[12]
		CCTAGAGCACTTCGTGTCCGCTT		

Primer sequences are shown in the 5′–3′ direction; bp = base pairs

Genomic deoxyribonucleic acid was extracted using a commercial extraction kit (Isolate II Genomic DNA Kit, Meridian Bioscience, London, United Kingdom) according to the manufacturer’s instructions. DNA concentration and purity were evaluated before amplification. PCR amplification was performed using MyTaq™ HS DNA Polymerase (Bioline Reagents Ltd., London, United Kingdom) in a final reaction volume of 25 µL according to previously validated protocols [11–16]. All assays were conducted as singleplex reactions.

Each PCR run included a positive control, a no-template negative control, and an internal amplification control. Amplicons were separated by 1.5% agarose gel electrophoresis and interpreted based on expected fragment sizes. Blood-based PCR was selected as a noninvasive screening approach; however, its sensitivity may be reduced in cases of low pathogen load, inappropriate sampling timing, prior antimicrobial therapy, or infections localized primarily within hepatic tissue.

### Hierarchical clustering analysis

Hierarchical agglomerative clustering was performed using a binary matrix of predefined ultrasonographic variables coded as present or absent. Jaccard distance was selected to quantify pairwise dissimilarity because it is appropriate for asymmetric binary datasets and emphasizes shared positive features. Clustering was performed using the average linkage method.

Dogs with >20% missing ultrasonographic variables were excluded to minimize bias. For the remaining cases, missing binary values were imputed with the modal value to preserve sample size, although this approach may introduce bias and should therefore be interpreted with caution. As a sensitivity assessment, clustering results were visually compared with complete-case patterns and were found to be broadly consistent.

The dendrogram was predefined to obtain three clusters corresponding to the hepatitis, hepatic lipidosis, and cirrhosis phenotypes. Alternative cluster solutions ranging from two to five clusters were explored; however, these showed lower clinical interpretability and did not meaningfully improve phenotype separation.

Cluster stability and structural consistency were assessed qualitatively through dendrogram inspection and feature-grouping consistency. Clustering findings were interpreted as exploratory and hypothesis-generating rather than confirmatory. Clinicopathologic variables were subsequently summarized across clusters to facilitate interpretation of phenotypic patterns.

All clustering analyses were performed using R software version 4.0.1 (R Foundation for Statistical Computing, Vienna, Austria). Hierarchical agglomerative clustering was used to explore co-occurrence patterns of clinical signs and ultrasonographic findings. Binary variables were coded as presence (1) or absence (0). Pairwise dissimilarities between dogs were calculated using the Jaccard distance implemented in the vegdist function of the vegan package (method = “jaccard”, binary = TRUE). Clustering was then conducted using the hclust function with the average-linkage method (method = “average”).

### Statistical analysis

Associations between host-associated factors and ultrasonographic positivity were evaluated using univariable logistic regression and reported as odds ratios (OR) with 95% confidence intervals (CI). Variables were initially screened using univariable analysis; however, multivariable modeling was not performed because of the limited sample size and sparse subgroup data. Therefore, all estimates should be interpreted as exploratory.

Body condition score was assessed using a standardized 9-point scale by the attending clinician. Breeds were categorized as exotic (purebred) or indigenous (local or mixed breed) for statistical analysis.

Proportions were calculated as the number of positive cases divided by the total number of dogs within the relevant category and were expressed as percentages. The 95% CI for proportions was estimated using the Wald approximation for binomial data and computed in R version 4.0.1. Because of the relatively small sample sizes in certain subgroups, some CI values were wide and should be interpreted with caution.

All statistical analyses, including logistic regression and clustering procedures, were performed using R software version 4.0.1. A p < 0.05 was considered statistically significant.

## RESULTS

### Case enrolment, ultrasonographic detection, and distribution of hepatic disorders

During January–November 2025, 1,278 dogs were presented to the Veterinary Teaching Clinic, Can Tho University, Vietnam. Of these, 111/1,278 dogs (8.69%; 95% CI: 7.27–10.31) were suspected of hepatobiliary disease and subsequently underwent abdominal ultrasonography. Liver abnormalities compatible with hepatobiliary disease were detected in 63/111 suspected dogs (56.76%; 95% CI: 47.53–65.55), corresponding to an overall detection proportion of 63/1,278 dogs (4.93%; 95% CI: 3.85–6.29). Table 4 summarizes the host-associated risk factor screening results for ultrasonographically confirmed liver disease among clinically suspected dogs.

**Table 4:** Risk factor screening for ultrasonographically confirmed liver disease among dogs suspected of hepatobiliary disease (n = 111).

Risk factor	Category	No. of examined	No. of positive	Positivity (%) [95% CI]	OR [95%CI (OR)]	p-value
Sex	Male	68	39	57.35 [45.50–68.60]	1.07 [0.49–2.30]	0.873
	Female	43	24	55.81 [41.00–69.88]	Ref.	
Breed	Mixed breed	72	46	63.89 [52.42–74.26]	2.29 [1.03–5.07]	0.039
	Vietnamese dog	39	17	43.59 [28.95–59.14]	Ref	
Age	>8 years	53	39	73.58 [60.69–83.98]	13.93 [2.71–71.55]	0.001
	>4–8 years	31	18	58.06 [40.62–74.08]	6.92 [1.29–37.05]	
	>1–4 years	15	4	26.67 [9.74–51.66]	1.82 [0.27–12.17]	
	≤1 year	12	2	16.67 [3.63–43.62]	Ref.	
Housing system	Free-roaming	70	43	61.43 [49.75–72.18]	1.67 [0.77–3.64]	0.194
	Confined	41	20	48.78 [34.00–63.72]	Ref.	
Body condition	Obese	50	34	68.00 [54.34–79.64]	4.78 [1.72–13.30]	0.007
	Underweight	35	21	60.00 [43.51–74.91]	3.38 [1.15–9.87]	
	Normal	26	8	30.77 [15.75–49.80]	Ref.	

CI = confidence interval, OR = odds ratio, ref.= reference category, Positivity (%) = number positive/ number examined × 100. 95% CI for proportions estimated using Jeffreys interval. ORs and 95% CI calculated using the reference group shown as “ref.” p-values indicate the overall association for each risk factor from univariable logistic regression. ORs are crude (univariable) estimates; no multivariable adjustment was performed.

No significant associations were identified for sex (p = 0.873) or housing system (p = 0.194). In contrast, breed, age, and body condition were associated with ultrasonographic positivity. Exotic breeds had higher odds of ultrasonographic positivity than indigenous breeds (OR, 2.29; 95% CI: 1.03–5.07). Compared with dogs aged ≤1 year, the odds of ultrasonographic positivity increased progressively with age, with the strongest associations observed in dogs aged >8 years (OR, 13.93; 95% CI: 2.71–71.55) and dogs aged 4–8 years (OR, 6.92; 95% CI: 1.29–37.05). Relative to dogs with normal body condition, obesity (OR, 4.78; 95% CI: 1.72–13.30) and underweight status (OR, 3.38; 95% CI: 1.15–9.87) were associated with increased odds of ultrasonographic positivity (p = 0.007).

Among dogs with ultrasonographic evidence of liver disease (n = 63), hepatitis was the most common imaging phenotype (36/63; 57.14%; 95% CI: 44.90–69.34%), followed by hepatic lipidosis (19/63; 30.16%; 95% CI: 18.86–41.46%) and cirrhosis (8/63; 12.70%; 95% CI: 4.50–20.90%) (Table 5).

**Table 5:** Distribution of ultrasonographically diagnosed hepatic disorders among dogs with ultrasonographic evidence of liver disease (n = 63).

Hepatic disorder	No. of dogs (n)	Proportion (%)	95% CI (%)
Hepatitis	36	57.14	44.90–69.34
Hepatic lipidosis	19	30.16	18.86–41.46
Cirrhosis	8	12.70	4.50–20.90

Values are presented as proportions (%) with 95% confidence intervals (CI) calculated using the Wald method. Percentages are based on the total number of dogs with ultrasonographic evidence of liver disease (n = 63).

### Clinical findings, ultrasonographic findings, clinicopathologic profile, and PCR etiologic screening in dogs with hepatitis (n = 36)

**Figure 1:**
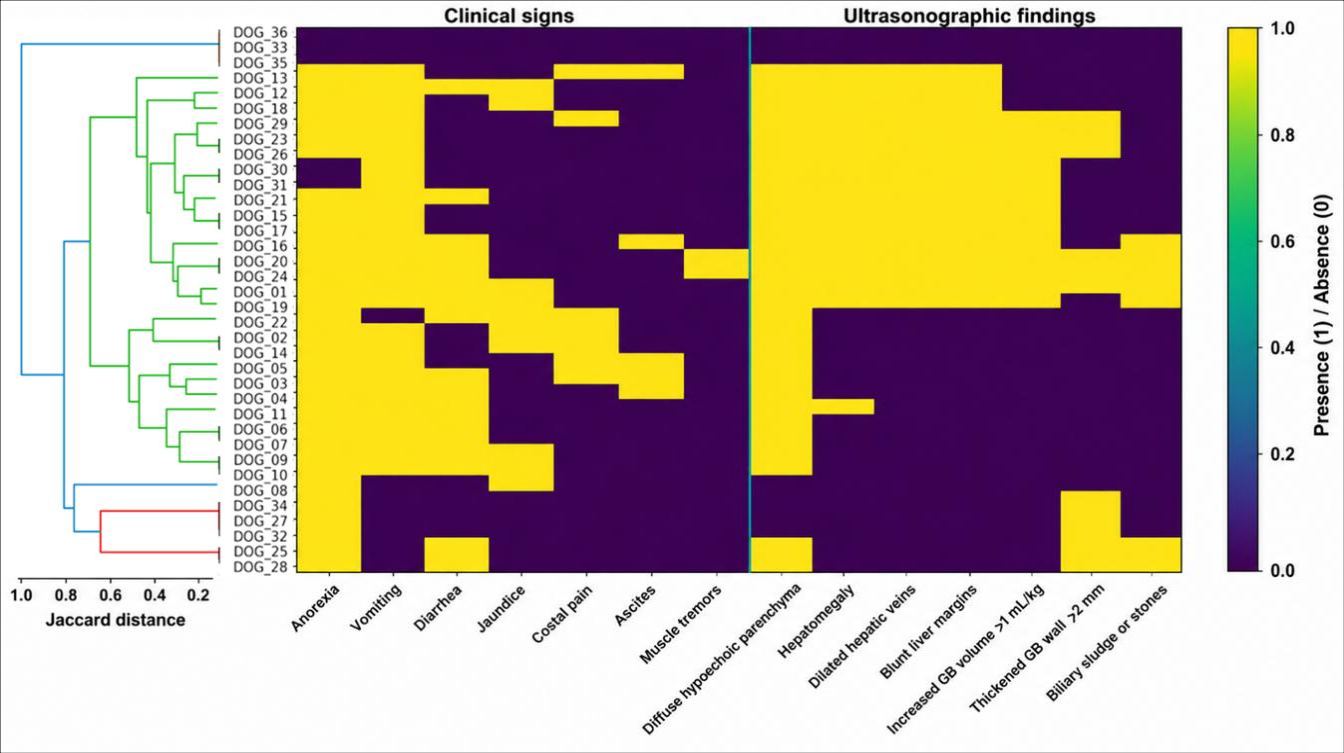
Integrated hierarchical clustering heatmap of combined clinical signs and ultrasonographic abnormalities in dogs with a hepatitis ultrasonographic phenotype (n = 36). Hierarchical clustering was performed using Jaccard distance and average linkage based on binary presence/absence data. Rows represent individual dogs and columns represent clinical and ultrasonographic variables. Dogs and variables are ordered according to dendrogram leaf position. Color intensity indicates feature presence or absence.

**Figure 2:**
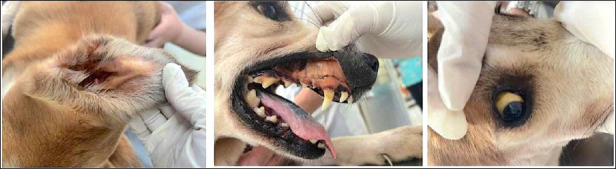
Representative clinical manifestations in dogs with a hepatitis ultrasonographic phenotype. (A) Auricular ecchymoses. (B) Ocular alterations and scleral icterus.

Clinical signs in dogs diagnosed with hepatitis were dominated by nonspecific gastrointestinal manifestations (Figures 1–2). Anorexia was the most frequent clinical sign (34/36; 94.44%), followed by vomiting (30/36; 83.33%) and diarrhea (18/36; 50.00%). Icterus was observed in 13 of 36 dogs (36.11%). Abdominal pain was recorded in 9/36 dogs (25.00%), ascites in 7/36 dogs (19.44%), whereas muscle tremors were uncommon (2/36; 5.55%).

**Figure 3:**
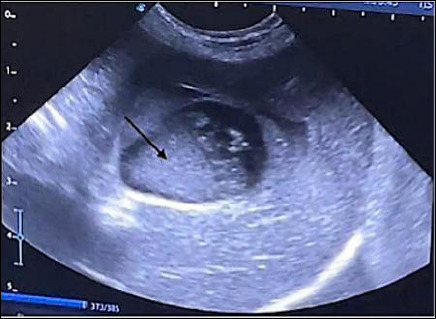
Representative ultrasonographic abnormality in a dog with a hepatitis ultrasonographic phenotype. The arrow indicates echogenic intraluminal material within the gallbladder consistent with biliary sludge.

**Figure 4:**
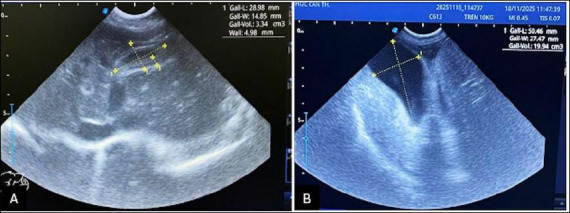
Abdominal ultrasonographic findings in a dog with a hepatitis ultrasonographic phenotype and concurrent cholecystitis. (A) Increased and heterogeneous hepatic echogenicity with gallbladder wall thickening (4.98 mm). (B) Gallbladder distension (VGB = 19.94 mL) with hepatomegaly, increased hepatic margin echogenicity, and mildly decreased hepatic parenchymal echogenicity.

**Figure 5:**
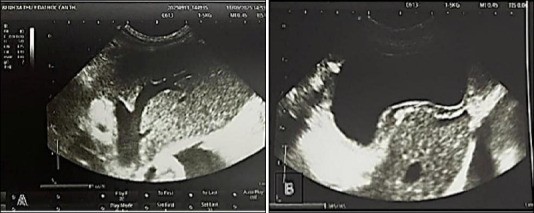
Representative ultrasonographic findings of hepatic vasculature and gallbladder abnormalities in a dog with hepatobiliary disease. (A) Dilated hepatic veins. (B) Gallbladder distension.

Ultrasonography revealed a consistent hepatobiliary pattern among dogs with hepatitis (Figures 1 and 3). Diffuse hepatic parenchymal hypoechogenicity was the predominant abnormality (32/36; 88.89%) and was frequently accompanied by hepatomegaly (19/36; 52.77%). Dilated hepatic veins and blunt hepatic margins were each detected in 18/36 dogs (50.00%). Gallbladder-associated abnormalities were common, including increased VGB (15/36; 41.70%) and gallbladder wall thickening (11/36; 30.56%). Biliary sludge or cholelithiasis was identified in 7/36 dogs (19.44%). Representative ultrasonographic images demonstrated hepatitis with concurrent biliary abnormalities, including heterogeneous hepatic echotexture with gallbladder wall thickening (4.98 mm) and gallbladder distension (VGB = 19.94 mL) (Figure 4), as well as hepatic venous dilation with gallbladder distension (Figure 5).

Hematologic and biochemical classifications are summarized in Table 6. Thrombocytopenia and increased hepatobiliary enzyme activities were frequently observed in dogs with hepatitis.

**Table 6:** Frequency of hematologic and serum biochemical abnormalities in dogs with hepatitis (n = 36).

Variable (unit)	Reference interval*	Category	Range	Mean ± SE	No. of dogs	Proportion (%)
RBC (×10⁶/µL)	5.5–8.5	Increased	8.65–9.34	8.95 ± 0.20	3	8.33
		Within reference	6.27–8.37	7.39 ± 0.24	10	27.78
		Decreased	1.38–5.49	3.79 ± 0.29	23	63.89
WBC (×10³/µL)	6–17	Increased	17.41–262	55.34 ± 13.38	24	66.67
		Within reference	8.32–16.23	11.21 ± 0.92	8	22.22
		Decreased	2.81–5.86	4.50 ± 0.63	4	11.11
PLT (×10³/µL)	200–900	Within reference	208–494	296.00 ± 51.56	5	13.89
		Decreased	3–176	91.65 ± 11.37	31	86.11
AST (U/L)	8.9–48.5	Increased	51–1,348	240.53 ± 48.09	32	88.89
		Within reference	24–42	35.00 ± 3.94	4	11.11
ALT (U/L)	8.2–57.3	Increased	63–1,112	212.27 ± 35.56	33	91.67
		Within reference	42–47	44.67 ± 1.45	3	8.33
ALP (U/L)	20–150	Increased	117–≥2,000	457.94 ± 79.46	33	91.67
		Within reference	20–90	46.00 ± 22.12	3	8.33
TBIL (mg/dL)	0.1–0.9	Increased	1.03–14.07	3.04 ± 0.49	27	75.00
		Within reference	0.23–0.41	0.29 ± 0.02	9	25.00
ALB (g/dL)	2.2–4.4	Within reference	2.10–4.00	3.03 ± 0.17	14	38.89
		Decreased	0.80–2.05	1.62 ± 0.08	22	61.11
GLO (g/dL)	2.3–5.2	Increased	5.30–9.10	6.64 ± 0.23	25	69.44
		Within reference	2.50–5.20	3.66 ± 0.28	11	30.56
CHOL (mg/dL)	110–320	Increased	328–563	450.88 ± 14.97	17	47.22
		Within reference	158–312	209.64 ± 16.89	11	30.56
		Decreased	86–109	96.63 ± 3.13	8	22.22

Mean ± SE = mean ± standard error, Range = observed range.

* Reference intervals are based on The Merck Veterinary Manual [10]. ALB = albumin, ALP = alkaline phosphatase, ALT = alanine aminotransferase, AST = aspartate aminotransferase, CHOL = cholesterol, GLO = globulin, PLT = platelet count, RBC = red blood cell count, TBIL = total bilirubin, WBC = white blood cell count.

PCR etiologic screening results for dogs with a hepatitis phenotype are summarized in Table 7. An infectious etiology was identified in 20/36 dogs (55.56%), including vector-borne pathogens (15/36; 41.67%), *Leptospira* spp. (3/36; 8.33%), and CAdV-1 (2/36; 5.56%). Dogs with positive PCR findings were excluded from the PCR-negative subgroup used for descriptive comparison. The PCR-negative subgroup comprised 16/36 dogs (44.44%) that tested negative for all PCR targets.

**Table 7:** Polymerase chain reaction (PCR)-based etiologic classification in dogs with a hepatitis ultrasonographic phenotype (n = 36).

PCR-based category	Etiologic target	No. positive (n)	Proportion (%)	95% CI (%)
Blood-parasite PCR positive	Any of targets below	15	41.67	25.50–57.83
	*Ehrlichia canis*	9	25.00	10.86–39.14
	*Anaplasma platys*	3	8.33	0.00–17.33
	*Babesia* spp.	2	5.56	0.00–13.06
	*Hepatozoon canis*	1	2.78	0.00–8.21
*Leptospira* spp. PCR positive	*Leptospira* spp.	3	8.33	0.00–17.33
Viral PCR positive	CAdV-1	2	5.56	0.00–13.06
All PCR negative	Negative to all above	16	44.44	28.22–60.56
Total		36	100	

Values are presented as proportions (%) with 95% confidence intervals (CI) calculated using the Wald method. Percentages are based on the total number of dogs with a hepatitis ultrasonographic phenotype undergoing PCR testing (n = 36). Individual pathogen percentages are calculated relative to the same denominator. PCR negative indicates no detectable target among all assays.

### Clinical presentation, ultrasonographic abnormalities, and clinicopathologic alterations in dogs with hepatic lipidosis (n = 19).

**Figure 6:**
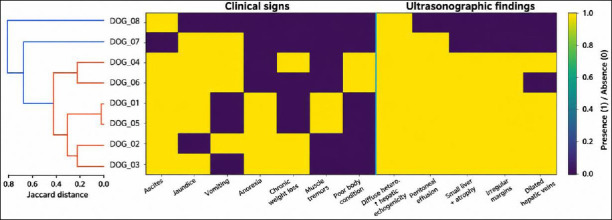
Distance-based hierarchical clustering heatmap of combined clinical signs and ultrasonographic abnormalities in dogs with hepatic lipidosis (n = 19). Hierarchical clustering was performed using Jaccard distance and average linkage based on binary presence/absence data. Rows represent individual dogs and columns represent clinical and ultrasonographic variables. Dogs and variables are ordered according to dendrogram leaf position. Color intensity indicates the presence or absence of a feature.

Clinical and ultrasonographic findings in dogs with hepatic lipidosis are summarized in Figure 6. The most frequent clinical manifestations were abdominal distension/discomfort (17/19; 89.47%), followed by inappetence (10/19; 52.63%) and diarrhea (10/19; 52.63%). Vomiting (7/19; 36.84%) and lethargy (6/19; 31.57%) occurred at intermediate frequencies, whereas jaundice was less common (4/19; 21.05%).

Ultrasonographically, hepatic lipidosis was characterized predominantly by diffuse parenchymal abnormalities, including increased hepatic echogenicity and hepatomegaly (each 13/19; 68.42%), reduced conspicuity of intrahepatic and portal vasculature (13/19; 68.42%), and increased liver-to-renal cortical echogenicity (12/19; 63.16%). Perihepatic and biliary-associated abnormalities were identified in subsets of dogs, including pericholecystic fat accumulation (10/19; 52.63%) and blunt hepatic margins with increased parenchymal brightness (8/19; 42.11%). In contrast, overt gallbladder abnormalities were relatively uncommon, with gallbladder wall thickening observed in 2/19 dogs (10.53%) and gallbladder enlargement in 1/19 dog (5.26%) (Figure 6).

**Figure 7:**
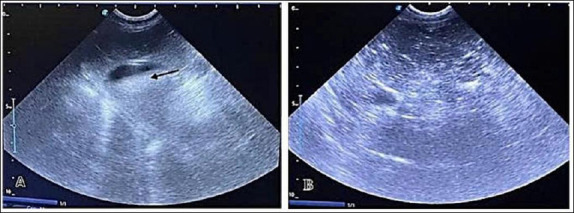
Ultrasonographic appearance of grade 3 hepatic lipidosis in a dog. (A) Diffuse increase in hepatic echogenicity; the arrow indicates a focal hyperechoic layer adjacent to the gallbladder wall consistent with pericholecystic fat. (B) Marked diffuse hepatic hyperechogenicity with reduced conspicuity of portal venous structures.

**Figure 8:**
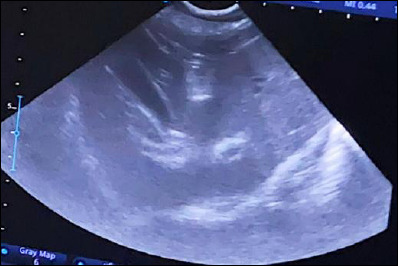
Ultrasonographic appearance of concurrent cholecystitis and grade 1 hepatic lipidosis in a dog. Gallbladder wall thickening with increased echogenicity and heterogeneous hepatic parenchyma containing focal hyperechoic areas.

Representative ultrasonographic images supported these phenotypes (Figures 7–8). Grade 3 hepatic lipidosis was demonstrated by diffuse hepatic hyperechogenicity, reduced visualization of portal venous structures, and a hyperechoic layer adjacent to the gallbladder wall, consistent with pericholecystic fat (Figure 7A–B). A dog with concurrent cholecystitis and grade 1 hepatic lipidosis exhibited gallbladder wall thickening with increased echogenicity and heterogeneous hepatic parenchyma containing focal hyperechoic areas (Figure 8). Hematologic and biochemical classifications are summarized in Table 8. Hematologic and biochemical classifications are summarized in Table 8.

**Table 8:** Frequency of hematologic and serum biochemical abnormalities in dogs with hepatic lipidosis (n = 19).

Variable (unit)	Reference interval*	Category	Range	Mean ± SE	No. of dogs	Proportion (%)
RBC (×10⁶/µL)	5.5–8.5	Increased	8.63–9.06	8.85 ± 0.22	2	10.53
		Within reference	5.79–7.08	6.48 ± 0.20	7	36.84
		Decreased	4.08–5.40	4.67 ± 0.16	10	52.63
WBC (×10³/µL)	6–17	Increased	17.29–172	43.83 ± 12.62	12	63.16
		Within reference	7.86–12.85	10.42 ± 1.02	5	26.31
		Decreased	5.72–5.73	5.73 ± 0.01	2	10.53
PLT (×10³/µL)	200–900	Within reference	126–503	311.50 ± 62.34	6	31.58
		Decreased	81–198	153.92 ± 10.56	13	68.42
AST (U/L)	8.9–48.5	Increased	50–1,035	167.40 ± 63.47	15	78.95
		Within reference	20–39	28.75 ± 4.23	4	21.05
ALT (U/L)	8.2–57.3	Increased	70–297	132.65 ± 17.65	17	89.47
		Within reference	41–47	44.00 ± 3.00	2	10.53
ALP (U/L)	20–150	Increased	205–≥2,000	611.18 ± 138.33	12	63.16
		Within reference	99–143	121.00 ± 22.00	7	36.84
TBIL (mg/dL)	0.1–0.9	Increased	1.02–7.48	2.74 ± 0.58	10	52.63
		Within reference	0.15–0.22	0.19 ± 0.01	9	47.37
ALB (g/dL)	2.2–4.4	Within reference	2.70–3.60	3.18 ± 0.14	8	42.11
		Decreased	0.90–2.00	1.46 ± 0.12	11	57.98
GLO (g/dL)	2.3–5.2	Increased	5.30–7.10	5.94 ± 0.19	9	47.37
		Within reference	2.40–4.70	3.51 ± 0.26	10	52.63
CHOL (mg/dL)	110–320	Increased	342–641	477.18 ± 31.51	11	57.98
		Within reference	139–233	183.38 ± 12.40	8	42.11

Mean ± SE, mean ± standard error; Range, observed range.

* Reference intervals are based on The Merck Veterinary Manual [10]. Abbreviations: ALB, albumin; ALP, alkaline phosphatase; ALT, alanine aminotransferase; AST, aspartate aminotransferase; CHOL, cholesterol; GLO, globulin; PLT, platelet count; RBC, red blood cell count; TBIL, total bilirubin; WBC, white blood cell count.

### Clinical presentation, ultrasonographic abnormalities, and clinicopathologic alterations in dogs with cirrhosis (n = 8).

**Figure 9:**
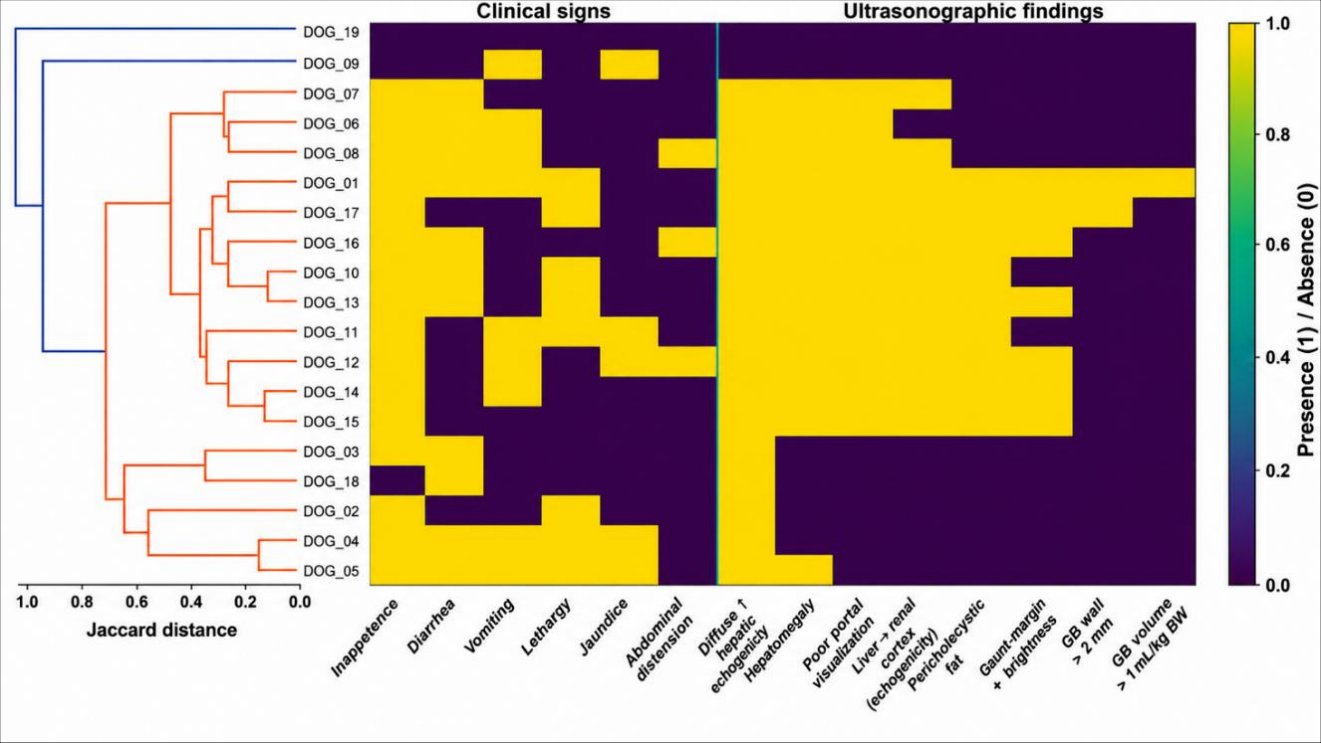
Distance-based hierarchical clustering heatmap of combined clinical signs and ultrasonographic abnormalities in dogs with cirrhosis (n = 8). Hierarchical clustering was performed using Jaccard distance and average linkage based on binary presence/absence data. Rows represent individual dogs and columns represent clinical and ultrasonographic variables. Dogs and variables are ordered according to dendrogram leaf position. Color intensity indicates feature presence or absence.

Hierarchical clustering analysis of dogs with cirrhosis identified two principal phenotypic patterns (Figure 9). One low-feature cluster exhibited relatively few concurrent abnormalities, including 1–2 clinical and ultrasonographic features, whereas the remaining dogs formed a higher-feature cluster characterized by multiple co-occurring clinical signs (4–5 features) and concordant ultrasonographic abnormalities (4–5 features), consistent with more advanced structural and portal hypertensive changes.

**Figure 10:**
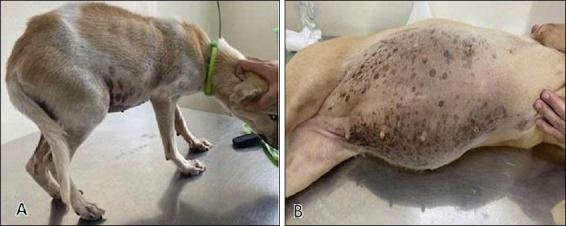
Representative clinical manifestations in dogs with cirrhosis. (A) Poor body condition with abdominal distension consistent with ascites. (B) Jaundice with marked abdominal distension consistent with ascites.

Ascites was the most frequent clinical manifestation (7/8; 87.50%), followed by jaundice (6/8; 75.00%). Vomiting and anorexia were each observed in 4/8 dogs (50.00%). Muscle tremors occurred in 3/8 dogs (37.50%), whereas chronic weight loss and poor body condition were each recorded in 2/8 dogs (25.00%) (Figure 9). Representative clinical appearances are presented in Figure 10.

**Figure 11:**
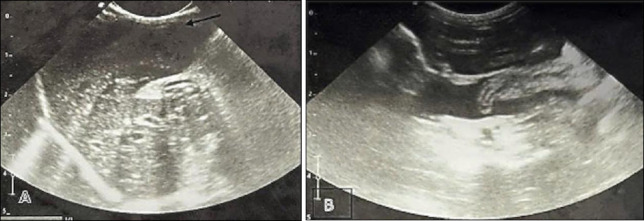
Ultrasonographic appearance in a dog with cirrhosis. (A) Increased echogenicity along the hepatic margin. (B) Abdominal (peritoneal) effusion.

**Figure 12:**
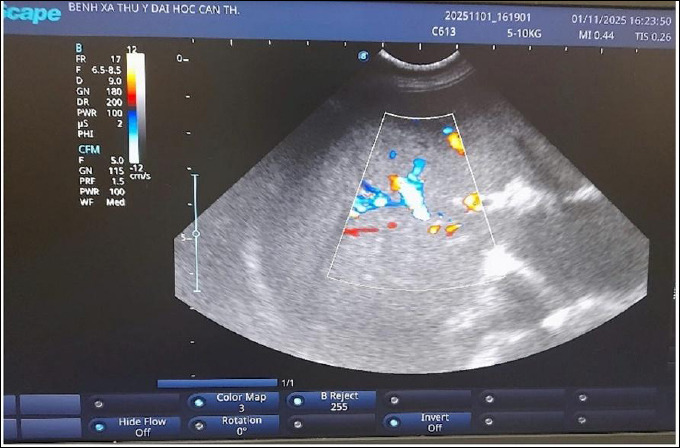
Color Doppler ultrasonography in a dog with cirrhosis demonstrating dilated hepatic veins. Color Doppler imaging demonstrates hepatic venous blood flow with increased vessel diameter compared with the expected normal caliber.

Ultrasonographically, diffuse heterogeneous hepatic echotexture was identified in all dogs with cirrhosis (8/8; 100%). Peritoneal effusion occurred in 7/8 dogs (87.50%). A small or atrophic liver and irregular hepatic margins were each observed in 6/8 dogs (75.00%), whereas dilated hepatic veins were identified in 5/8 dogs (62.50%) (Figure 9). Representative ultrasonographic images demonstrated increased hepatic edge echogenicity with abdominal effusion and color Doppler evidence of hepatic venous dilation (Figures 11–12). Hematologic and biochemical classifications are summarized in Table 9.

**Table 9:** Frequency of hematologic and serum biochemical abnormalities in dogs with cirrhosis (n = 8).

Variable (unit)	Reference interval*	Category	Range	Mean ± SE	No. of dogs	Proportion (%)
RBC (×10⁶/µL)	5.5–8.5	Increased	9.39–10.05	9.72 ± 0.33	2	25.00
		Within reference	7.29–7.76	7.53 ± 0.24	2	25.00
		Decreased	1.76–4.27	3.12 ± 0.52	4	50.00
WBC (×10³/µL)	6–17	Increased	19.36–47.79	29.80 ± 6.30	4	50.00
		Within reference	8.93–10.39	9.66 ± 0.73	2	25.00
		Decreased	2.05–3.48	2.77 ± 0.72	2	25.00
PLT (×10³/µL)	200–900	Within reference	235–294	264.50 ± 29.50	2	25.00
		Decreased	2.10–104	51.52 ± 20.02	6	75.00
AST (U/L)	8.9–48.5	Increased	58–256	127.50 ± 30.99	6	75.00
		Within reference	34.00–47.70	40.85 ± 6.85	2	25.00
ALT (U/L)	8.2–57.3	Increased	63–165	99.83 ± 15.35	6	75.00
		Within reference	35–43	39.00 ± 4.00	2	25.00
ALP (U/L)	20–150	Increased	166–282	206.00 ± 20.77	5	62.50
		Within reference	50–56	52.00 ± 2.00	3	37.50
TBIL (mg/dL)	0.1–0.9	Increased	1.02–2.78	1.53 ± 0.33	5	62.50
		Within reference	0.21–0.44	0.29 ± 0.08	3	37.50
ALB (g/dL)	2.2–4.4	Within reference	3.10–3.60	3.37 ± 0.15	3	37.50
		Decreased	1.50–2.10	1.76 ± 0.12	5	62.50
GLO (g/dL)	2.3–5.2	Increased	6.30–9.10	7.60 ± 0.40	6	75.00
		Within reference	3.20–4.50	3.85 ± 0.65	2	25.00
CHOL (mg/dL)	110–320	Within reference	223–234	233.50 ± 0.50	2	25.00
		Decreased	74–107	89.33 ± 4.45	6	75.00

Mean ± SE, mean ± standard error; Range, observed range.

* Reference intervals are based on The Merck Veterinary Manual [10]. Abbreviations: ALB, albumin; ALP, alkaline phosphatase; ALT, alanine aminotransferase; AST, aspartate aminotransferase; CHOL, cholesterol; GLO, globulin; PLT, platelet count; RBC, red blood cell count; TBIL, total bilirubin; WBC, white blood cell count.

## DISCUSSION

This prospective hospital-based study provides, to our knowledge, the first clinic-based evaluation in the Mekong Delta region of Vietnam integrating standardized ultrasonographic phenotyping with clinicopathologic profiling and blood-based PCR screening for infectious hepatobiliary disease in dogs. Over the 11-month study period, 8.69% of dogs were clinically suspected, and 4.93% were ultrasonographically confirmed to have hepatobiliary abnormalities, with hepatitis representing the predominant imaging phenotype. These findings provide updated regional data from tropical Vietnam and Southeast Asia, where infectious hepatopathies are common and advanced diagnostic procedures such as liver biopsy are often unavailable, and further support the use of ultrasonography as a pragmatic first-line triage tool in clinical practice.

### Host-associated factors linked with ultrasonographic positivity

Among clinically suspected dogs, ultrasonographic positivity increased with age and was associated with breed and extremes of body condition, whereas sex and housing system were not associated. The age-related increase, particularly in dogs aged >8 years, is consistent with the established epidemiology of chronic hepatopathies in small animal practice [17]. The association observed in exotic breeds may reflect referral bias or a potential genetic predisposition; however, this finding should be interpreted with caution because population-level denominators were unavailable. The observed association with both obesity and underweight status highlights the importance of metabolic imbalance and systemic disease in the clinical context of hepatobiliary disorders [17, 18].

### Limitations of imaging-based phenotypic classification

An important limitation of this study is the reliance on ultrasonographic phenotyping without histopatho-logical confirmation. Consequently, overlap among imaging findings and potential misclassification are likely and should be carefully considered when interpreting the findings. Chronic hepatitis may be particularly difficult to distinguish from early-stage hepatic fibrosis or reactive hepatopathy because these conditions frequently share overlapping ultrasonographic characteristics, including heterogeneous echotexture, altered echogenicity, and variable liver size. Early fibrotic remodeling may not yet produce the architectural distortion or nodular surface irregularity characteristic of advanced cirrhosis, thereby limiting the diagnostic specificity of ultrasonography. Therefore, differentiation between inflammatory and early fibrotic liver disease remains inherently constrained when based solely on imaging findings, and histopathology remains the gold standard for definitive diagnosis and disease staging [17–21]. These considerations indicate that the phenotypic classifications described in this study should be interpreted as clinically pragmatic rather than diagnostically definitive.

### Clinical relevance of ultrasonographic phenotypes

The distribution of ultrasonographic phenotypes (hepatitis > hepatic lipidosis > cirrhosis) reflects the spectrum of cases commonly referred for imaging evaluation. Importantly, these categories should be interpreted as imaging-supported clinical phenotypes rather than etiologic diagnoses, because ultrasonography is primarily effective at identifying structural abnormalities and associated complications but has limited specificity in differentiating underlying causes. This limitation is particularly relevant when distinguishing chronic hepatitis from early fibrotic liver disease, which may demonstrate similar ultrasonographic appearances in routine clinical practice. In selected cases, advanced imaging modalities may provide complementary diagnostic information, particularly in complex hepatobiliary or biliary disease presentations [19].

### Hepatitis phenotype and infectious etiologic screening

Dogs classified with the hepatitis phenotype most commonly exhibited nonspecific gastrointestinal signs together with diffuse hepatic parenchymal hypoechogenicity, frequently accompanied by hepatomegaly and concurrent gallbladder abnormalities. These findings are consistent with previous descriptions of inflammatory hepatopathies, in which hepatocellular injury and cholestasis are reflected in both ultrasonographic and biochemical alterations [7, 17, 20, 22]. However, gallbladder wall thickening should be interpreted with caution because it may also occur under physiologic conditions, such as fasting, or in systemic disorders, such as hypoalbuminemia, in which edema rather than primary biliary inflammation is the underlying mechanism [23, 24].

A major strength of the present study is the incorporation of PCR-based screening for selected infectious etiologies, including blood-borne pathogens, *Leptospira* spp., and CAdV-1, in dogs classified with the hepatitis phenotype. This integrated approach reduces, although does not eliminate, the risk of misclassifying acute infectious hepatitis as chronic hepatopathy, particularly considering the possibility of false-negative PCR results associated with low pathogen load, inappropriate timing of sample collection, or previous antimicrobial therapy [25–27].

### Ultrasonographic characteristics of hepatic lipidosis and cirrhosis

Dogs with hepatic lipidosis demonstrated characteristic ultrasonographic findings, including diffuse hyperechogenicity, hepatomegaly, and reduced visualization of intrahepatic vasculature, consistent with lipid infiltration altering acoustic interfaces [7, 25, 29]. Clinicopathologic findings further supported a metabolic component, with frequent alterations in hepatocellular enzyme activities and lipid metabolism [28].

In contrast, dogs with cirrhosis exhibited ultrasonographic and clinical findings consistent with advanced chronic liver disease, including ascites, heterogeneous hepatic echotexture, atrophic liver morphology, and vascular abnormalities suggestive of portal hypertension. These findings align with previous reports and reinforce the clinical relevance of ultrasonography in identifying late-stage structural alterations and associated complications [7, 20, 21, 29, 30].

### Interpretation of hierarchical clustering analysis

The hierarchical clustering analysis provided an additional exploratory framework for assessing co-occurrence patterns among clinical and ultrasonographic abnormalities. The use of Jaccard distance for binary variables was methodologically appropriate; however, the number of clusters was predefined according to clinical phenotypes, and alternative cluster solutions were not formally optimized using cluster-validity indices. Consequently, these findings should be interpreted as descriptive and hypothesis-generating rather than confirmatory.

### Study limitations and future perspectives

Several additional limitations should be acknowledged. The single-center, referral-based study design may introduce selection bias and limit generalizability to primary-care canine populations. The relatively small sample size of the cirrhosis subgroup (n = 8) reduced statistical power for subgroup comparisons. In addition, the absence of multivariable analysis limited the ability to control for potential confounding variables; therefore, the reported associations should be interpreted as exploratory. Furthermore, PCR-negative findings do not exclude infection, as detection may be influenced by pathogen load, sampling timing, prior antimicrobial treatment, and tissue-localized infections that may not be detectable in peripheral blood samples.

Despite these limitations, the present study demonstrates that an integrated diagnostic approach combining ultrasonography, clinicopathologic assessment, and targeted PCR screening can provide a practical and clinically applicable framework for the initial evaluation and triage of canine hepatobiliary disease. Future multicenter studies incorporating histopathology and expanded etiologic diagnostic testing are warranted to validate these findings, reduce potential misclassification, and improve diagnostic accuracy and disease characterization.

## CONCLUSION

Canine hepatobiliary disease accounted for a measurable proportion of the referral caseload at the Veterinary Teaching Clinic, Can Tho University, Vietnam, with ultrasonographic abnormalities identified in 4.93% of all canine presentations and 56.76% of clinically suspected dogs. Hepatitis was the predominant ultrasono-graphic phenotype, followed by hepatic lipidosis and cirrhosis. Dogs with hepatitis frequently demonstrated diffuse hepatic hypoechogenicity, hepatomegaly, and concurrent gallbladder abnormalities, whereas hepatic lipidosis was characterized primarily by diffuse hyperechogenicity and altered intrahepatic vascular conspicuity. Cirrhosis was associated with advanced structural alterations, including ascites, heterogeneous hepatic echotexture, hepatic atrophy, and vascular abnormalities suggestive of portal hypertension.

Older age and abnormal body condition were significantly associated with increased odds of ultrasonographic positivity, highlighting the importance of host-associated factors in canine hepatobiliary disease. In addition, PCR screening identified infectious agents in more than half of dogs with a hepatitis phenotype, supporting the potential contribution of vector-borne pathogens, *Leptospira* spp., and CAdV-1 to hepatobiliary abnormalities in dogs from tropical environments.

A major strength of this study was the integration of ultrasonography, clinicopathologic assessment, and targeted PCR screening within a prospective clinical framework. This combined diagnostic approach provided a practical, clinically applicable strategy for the preliminary evaluation and triage of hepatobiliary disease in resource-limited veterinary settings, where histopathology and advanced diagnostic modalities are not routinely available.

Overall, this study demonstrates that ultrasonography combined with clinicopathologic evaluation and targeted PCR screening can serve as an effective preliminary diagnostic framework for canine hepatobiliary disease in tropical clinical practice. Future multicenter investigations incorporating histopathology, expanded etiologic testing, and larger study populations are warranted to validate these findings, improve phenotypic classification, and enhance diagnostic accuracy and clinical management of canine hepatobiliary disorders.

## DATA AVAILABILITY

All datasets generated and analyzed during the study are included in the manuscript.

## OFF-LABEL ANTIMICROBIAL DECLARATION

Some animals in this study received off-label antimicrobial therapy, including azithromycin, doxycycline, ampicillin, ampicillin–sulbactam, and metronidazole.

## AUTHORS’ CONTRIBUTIONS

TTT: Conceptualization, study design, supervision, data interpretation, writing – original draft, and writing – review and editing. NTPC: Data curation, formal analysis support, and writing – review and editing. TNB: Data curation and writing – review and editing. All authors read and approved the final manuscript.
